# Surgery

**Published:** 1893-06

**Authors:** 


### SURGERY.

EDITED BY DR. F. W. McRAE.

---

### AN IMPROVED TECHNIQUE IN THE OPERATION OF EXTERNAL PERINEAL URETHROTOMY.

In the *New York Medical Journal,* January 21, 1893, Fluhrer describes his method of performing the operation of external perineal urethrotomy. When a filiform bougie can be introduced through the stricture he passes over it a urethrotome resembling that of Teevan, and divides all strictures anterior to the perineum. The urethrotome being withdrawn, a Gouley tunnelled staff is passed over the guide and, when the stricture is reached, it is reversed so that its beak projects toward the surface of the perineum; on it a small incision is made into the urethra. A thread is then attached to each side of the urethral opening to act as retractors, and the portion of the guide in the penile urethra withdrawn through the opening. A small hook is next fitted into the extremity of the staff, and it is used to draw upward the incision in the urethra. By this means and the two threads the urethra is fully exposed. The urethrotome being threaded again on the guide, is introduced into the bladder and the stricture cut on the floor. Further incisions may be made on a director introduced into the bladder through the wound. If the stricture is impermeable, then endeavor to feel the urethra behind, and cut forward or open at the apex of the prostate after the method of Cock.—*Univ. Med. Mag.*

---

### THE MANAGEMENT OF SUPPURATION COMPLICATING TUBERCULOUS DISEASE OF THE BONES AND JOINTS.

In the *Medical and Surgical Reporter*, February 18, 1893, Gibney gives the following summary of his method of treating suppurative troubles affecting the bones and joints:

(1) Protect the joint about which the bone lesion exists in the early stage and in the latter stage, whether the abscess is let alone, aspirated or incised.

(2) In cases where the suppurative process is confined to a small area, it is good surgery to leave the small abscesses alone if the protective appliance is adequate.

(3) It is good practice to aspirate where the abscess is in the way of the proper adjustment of apparatus, and by such procedure one may expect good results in at least 50 per cent. of the cases aspirated.

(4) The simple incision of an abscess dependent upon bone disease depends for good result upon the extent of the bone lesion.

(5) Excision of the hip is not a measure to be employed in all cases where extensive suppuration exists, but must depend largely upon the condition of the patient and the location and extent of the abscesses.

(6) Expectant treatment for the knee and ankle joint in children yields the best results for life and limb.

(7) Amputation of the ankle in a child is rarely ever justifiable, except when amyloid disease of the liver or kidneys threatens or is present; of a hip after a thorough excision has failed.

(8) The long-continued employment of a good-fitting splint to the back in Pott's disease of the spine will yield better results than any operative procedures on the bone with which I am familiar.—*Univ. Med. Mag.*

---

### APPENDICITIS APHORISMS.

Dr. Robt. T. Morris, of New York, formulates the following aphorisms:

Statistics show that an appendicitis patient may be expected to recover several times, and to die once.

The appendix vermiformis should be removed as soon as a diagnosis of the appendicitis is made, because no one can tell at just what moment necrosis is going to follow the disturbance of circulation.

It matters not whether the swelling which cuts off the circulation of the appendix is caused by the usual catarrhal process, or by fecal concretions, or by foreign bodies. The mucous tube cannot swell readily within its inelastic sheath, so it chokes itself to death at any hour in the course of any attack.

The operation should be done at any stage of the disease in which the surgeon finds the patient, because the surgeon's methods are certain and safe, and nature's methods are uncertain and risky—speaking by comparison.

In patients who have had recurring appendicitis the appendix should be removed, even if there have been no recent attacks, because trouble may come when the patient is on the ocean or in the woods, away from skilled surgical help. Those who object to the proposition have never seen the horrible, black, stinking, ragged little crater that suddenly begins to erupt among an ususpecting patient's vitals.

An appendix that has once been inflamed, is a disabled appendix when adhesions bend it or compress it, because it cannot again swell evenly.

If the physician thinks that his patient is in luck because an appendicitis abscess has opened into the intestine and discharged its contents, he is badly mistaken. The rotting *dèbris* that remains is likely to set up a dangerous septicæmia, unless the surgeon opens up the tract from another direction and puts the poison all into a pail.—*Practice.*

